# Patient Factors in Inappropriate Antibiotic Prescribing for Upper Respiratory Tract Infection in the Emergency Department

**DOI:** 10.21315/mjms2021.28.2.7

**Published:** 2021-04-21

**Authors:** Azmi Ahmad, Junainah Nor, Ariff Arithra Abdullah, Tuan Hairulnizam Tuan Kamauzaman, Mohd Boniami Yazid

**Affiliations:** Department of Emergency Medicine, School of Medical Sciences, Universiti Sains Malaysia, Kubang Kerian, Kelantan, Malaysia

**Keywords:** antibiotic prescribing, emergency department, respiratory tract infections

## Abstract

**Background:**

Emergency departments (EDs) are frequently misused for non-emergency cases such as upper respiratory tract infections (URTIs). Flooding of these cases may contribute to inappropriate antibiotic prescribing. The aim of this study was to determine the patient factors associated with inappropriate antibiotic prescribing for URTIs in the EDs.

**Methods:**

This cross-sectional study involved patients over age 3 years old who presented with URTI to the green zone of the ED of a tertiary hospital on the east coast of Malaysia in 2018–2019. Convenient sampling was done. The patients were categorised into two groups according to their McIsaac scores: positive (≥ 2) or negative (< 2). Antibiotics given to the negative McIsaac group were considered inappropriate.

**Results:**

A total of 261 cases were included — 127 with positive and 134 with negative McIsaac scores. The most common symptoms were fever and cough. About 29% had inappropriate antibiotic prescribing with a high rate for amoxycillin. Duration of symptoms of one day or less (OR 18.5; 95% CI: 1.65, 207.10; *P* = 0.018), presence of chills (OR 4.36; 95% CI: 1.13, 16.88; *P* = 0.033) and diagnosis of acute tonsillitis (OR 5.26; 95% CI: 1.76, 15.72; *P* = 0.003) were significantly associated with inappropriate antibiotic prescription.

**Conclusion:**

Factors influencing inappropriate antibiotic prescribing should be pointed out to emergency doctors to reduce its incidence.

## Introduction

Upper respiratory tract infections (URTIs) or common colds are defined as infections that affect the nose, sinuses, pharynx and larynx with no indication of pneumonia. URTIs are among the most common presentations in emergency departments (EDs). Despite not being emergencies, a high number of URTI cases are unavoidable as EDs operate 24 h a day. A retrospective study done in the USA in 2014 showed that there were 126 million ED visits with URTI between the year 2001 and 2010, accounting for 12.2% of ED visits (a rate of 122 per 1,000 visits) (1).

The high incidence of URTI cases, time pressure due to overcrowding, limited support resources and staffing, and fear of missed diagnosis in the ED setting may influence inappropriate antibiotic prescribing. Besides creating a heavy burden on our healthcare system, this practice promotes antibiotic resistance. At least 30% of antibiotic courses prescribed were unnecessary (2). Most of these unnecessary prescriptions were for acute respiratory conditions such as colds, bronchitis and sore throat that are predominantly viral in origin. Despite studies showing a lack of clinical benefit from antibiotic use, irrational antibiotic prescribing was still reported to be high (3). A variety of factors affecting antibiotic prescribing behaviour included the sociodemographics of patients, clinical characteristics, physician judgement and local patterns of practice. To date, many studies have been carried out to identify the causative factors contributing to inappropriate antibiotic prescribing for URTI, mostly in primary healthcare institutions rather than in emergency settings. Although the ED caters to emergencies, it is frequently flooded with non-emergency cases. Usually treated in outpatient clinics, URTI patients often misuse the ED. Due to the unique nature of the ED, we decided to do our study in the ED, since the findings may be different from those conducted in outpatient department (OPD), which have a different working environment. Our study aimed to determine the patients’ presentation factors that may have influenced inappropriate antibiotic prescribing for URTI in ED settings. The most common presentation for URTI is sore throat, in which 80% are viral in origin, while only 15% of cases are caused by Group A beta-haemolytic streptococcus (GABHS). Due to possible serious complications with GABHS, such as acute rheumatic fever, rheumatic disease and peritonsillar abscess, many clinicians may prescribe antibiotics in URTI despite being viral in origin, thereby promoting inappropriate antibiotic prescribing. On this basis, several clinical decisions have been developed to aid clinicians in antibiotic prescribing for URTI patients. The McIsaac clinical scoring scale is a validated tool that predicts GABHS infection in URTI and thus helps reduce inappropriate antibiotic prescribing (4). Studies have shown that the McIsaac score has high sensitivity and acceptable specificity (5). A study done at the national level showed a significant reduction in antibiotic usage after applying the McIsaac rule for the paediatric population (6).

## Methods

This observational, cross-sectional study was conducted from November 2018 to August 2019 in the ED of a suburban, tertiary hospital on the east coast of Malaysia. All URTI patients who presented to the ED were referred from the triage area. The criteria included presenting with complaint of URTI symptoms (cough, runny nose, sore throat and fever) noted in the emergency triage and also a final diagnosis of URTI in the green zone’s registration book. Inclusion criteria were all URTI cases that were sent to the green zone. Those aged less than three years old and cases that were treated in yellow or red zones were excluded. This was because streptococcal throat infection is uncommon in patients under 3 years old, and patients treated in other zones have critical and semi-critical illnesses that may require further evaluation and investigation. Convenient sampling was done. Samples were taken from all URTI patients on Sunday and Monday from November 2018 to January 2019 and from May to June 2019. Samples were taken on Friday and Saturday from February to April 2019 and from July to August 2019. Samples were collected until the sample size was achieved.

All required information was taken from the standardised emergency clerking sheet. Data collected were evaluated for correct diagnosis and calculated for McIsaac score after the investigators reviewed the emergency clerking sheet. To protect patient confidentiality, a unique study ID was assigned to each patient from a pseudonym list containing relevant identification data and were only accessible to the investigator and his team.

## Instruments

The McIsaac score is a clinical scoring scale to help distinguish GABHS from viral pharyngitis and guides antibiotic therapy in the treatment of acute pharyngitis (4). Absence of cough, presence of tonsillar exudates, fever and tender anterior cervical nodes are given one point each. One point is added for those under 15 years old, while one point is deducted for those over 45 years old. In the original McIsaac score, no antibiotic treatment is advised for those with total scores of 0 and 1. Those with a total score of 2 should be treated empirically if the rapid test is positive, while those with 3 or 4 points are advised to receive antibiotic therapy. In our study, participants were divided into two groups — those with positive (≥ 2) or negative (< 2) McIsaac scores. Antibiotics given in the negative McIsaac group were considered inappropriate antibiotic prescribing as antibiotic treatment was not indicated in this group.[Fig f1-07mjms2802_oa4][Fig f2-07mjms2802_oa4]

## Statistical Analysis

Data were explored and analysed using Statistical Package for the Social Sciences (SPSS) version 25.0 (SPSS, Inc., Chicago, IL, USA). Numerical variables were presented using mean and standard deviation. Categorical variables were presented as frequency and percentage. Simple and multiple logistic regression were applied to determine patient factors associated with inappropriate antibiotic prescribing. Statistical significance was established as a *P*-value of < 0.05.

## Results

A total of 266 participants were selected for this study. Five patients were excluded for ages under 3 years old. Among the remaining 261 participants, 127 (48.7%) had a positive McIsaac score (≥ 2) while 134 (51.3%) had a negative McIsaac score (< 2). The mean ages were 11.93 years (SD = 11.68) and 32.54 years (SD = 19.40) in the positive and negative McIsaac groups, respectively. The participants’ ages in the positive McIsaac group ranged from 3 to 85 years old compared to 3 to 65 years old for the negative McIsaac group. Our study showed that a higher number of URTI patients sought treatment during the evening shift, which was 119 (45.6%), followed by 78 in the night shift (29.9%) and 64 in the morning shift (24.5%). Most patients had no previous visits for the same complaints in our centre or other healthcare facilities (204 patients or 78.2%), followed by second visit 50 (19.2%), and third visit 7 (2.7%). The characteristics of the patients are presented in Table 1.

A total of 102 (80.3%) patients in the positive McIsaac group were prescribed antibiotics compared to 76 (56.7%) patients in the negative McIsaac group. The proportion of patients who were inappropriately prescribed antibiotics (based on a negative McIsaac score) was 29.1% (76 of 261 patients). Both groups showed higher numbers in amoxycillin and amoxicillin/clavulanate acid as the choice of antibiotics (Table 2).

For statistical analysis, variables in sociodemographic, symptoms and signs were examined with simple logistic regression analysis (Table 3). Variables of working shift, duration of illness, presence of chills, sore throat and final diagnosis of tonsillitis showed a significant association with inappropriate antibiotic prescribing. Other variables such as age, gender, frequency of visits, cough, rhinitis, muscle pain, presence of cervical lymph nodes and tonsillar enlargement showed no significant association. For the final model, variables with a *P*-value < 0.25 were selected for multiple logistic regression. The results are presented in Table 4. A duration of symptoms of one day or less (OR 18.5; 95% CI: 1.65, 207.10; *P* = 0.018), presence of chills (OR 4.36; 95% CI: 1.13, 16.88; *P* = 0.033) and a diagnosis of tonsillitis (OR 5.26, 95% CI: 1.76, 15.72; *P* = 0.003) were significantly associated with inappropriate antibiotic prescribing.

## Discussion

URTI is the most common reason for consultation in health facilities and for antibiotic prescribing (7). Since antibiotic therapy is largely ineffective in the treatment of viral infection, which is its most common aetiology, this may lead to unnecessary use of antibiotics (8). Inappropriate antibiotic prescribing will not improve patient outcomes but may increase antimicrobial resistance, thus threatening our ability to treat infectious disease successfully. Persistently high rates of inappropriate antibiotic prescribing are likely to be multifactorial, ranging from patients’ sociodemographic data, signs and symptoms at presentation, patients’ expectations, and prescriber factors.

In our study, the proportion of patients who were inappropriately prescribed antibiotics was 29.1%. This finding was similar to a study by Fleming-Dutra et al. (2), which estimated that antibiotic prescription might have been inappropriate in about 30% of outpatients. On a national scale, our finding was lower compared to another local hospital, with 39.7% of inappropriate antibiotic prescribing (9).

In terms of patient demography, our study showed no significant association between gender and inappropriate antibiotic prescribing. This finding was consistent with a previous study done in Canada by Cadieux et al. (10) involving an analysis of almost 130,000 drug prescriptions for viral respiratory infections. Another study by Smith et al. (11) based on data from National Ambulatory Medical Care Survey (NAMCS) and National Hospital Ambulatory Medical Care Survey (NHAMCS) OPD showed similar findings. In our study, age showed no significant association with inappropriate antibiotic prescribing. Compared to previous studies, adults with acute cough were more likely to be prescribed antibiotics (12, 13). The probable explanation for this is that the prescribers believed that adults who came to the ED usually presented with infections of bacterial origin. With regard to racial differences, no significant result was observed considering that the majority of our patients were Malays (96.6%), with only 3.1% of patients from other ethnicities due to the Kelantan state being a predominantly Malay community (14).

Our study showed that a higher number of patients were treated for URTI during the evening shift (45.6%). We found that 64.5% (40 of 62 participants with negative McIsaac) had inappropriate antibiotic prescribing, and this was significantly higher than those treated during the morning shift. Since most health clinics are closed after office hours, this would cause an influx of patients to the ED for non-emergency cases. An increased number of patients and overcrowding may lead to inappropriate antibiotic prescribing. A previous study conducted in a tertiary care hospital on the east coast of Peninsular Malaysia involving 549 children showed no association between time of visit and antibiotic prescribing; in that study, the time of visit was categorised into two items — office hours (08:00–17:00) and non-office hours (17:01–07:59). Their study also showed that 198 (80%) of antibiotic prescribing occurred during non-office hours, which were further divided into two more items — evening and night time (15).

On the effect of the duration of illness prior to presentation to the ED, our study showed that patients with less than a one-day duration of symptom, which is suggestive of being viral in origin, were at a higher risk of being prescribed antibiotics. Since antibiotics are reserved for bacterial infections, this finding is in contrast with the current recommendation. A previous study suggested that bacterial URTI should be suspected in cases of unresolved symptoms after 10 days, severe onset with a concurrent temperature higher than 38 °C, and purulent nasal discharge for three consecutive days (16). Potential causes for this contradicting finding may be attributed to a few factors. Patient factors such as lack of knowledge regarding the disease, fear of severe illness, patients’ expectation and demand, as well as misconceptions about antibiotic effectiveness in treating viral URTI may put pressure on the attending doctors to prescribe antibiotics. A previous study showed that 50% of patients had pre-visit expectations for antibiotic prescription when presenting with URTI symptoms in primary healthcare facilities (17, 18). In addition, most prescribers in our green zone were junior doctors. With limited experience, diagnosis uncertainty and lack of communication skills to provide evidence-based medicine to the patients may have led them to prescribe antibiotics unnecessarily. Another factor to be considered is the designation or specialty of the attending doctor. A retrospective cross-sectional study by Ahmed et al. (19) showed that emergency physicians had a higher magnitude of prescribing antibiotics compared to paediatricians.

Based on simple logistic regression analysis, there was no significant association between the presence of fever and antibiotic prescribing for URTI. This result was inconsistent with the findings of Linder and Singer (20). In their study, 273 samples were evaluated and showed a strong association between fever and antibiotic prescribing (46% versus 29% antibiotic prescribing for those without charted fever; *P* < 0.005). Another retrospective study involving 1,662 veterans with respiratory infections showed that fever was a significant predictor for inappropriate antibiotic prescribing (21).

In our study, a presenting symptom of chills was significantly associated with inappropriate antibiotic prescribing for URTI in our ED. This finding may reflect that the prescribers associate chills with bacterial illness or were pressured to satisfy the patient’s expectation that chills should be perceived as severe and warranting antibiotics. Working in the ED, which has the highest number of non-emergency cases combined with limited resources and time to treat the patients, might have put greater pressure on prescribers to start antibiotics unnecessarily. A study by Butt et al. (22) showed that emergency physicians had a higher rate of inappropriate prescribing (74%), perhaps due to illnesses presenting in acute settings being perceived as severe. Combined with a lack of proper follow-up after discharge, this might have caused fear of complications and medicolegal issues, and cases were thus judged to warrant antibiotics (22).

As compared to the diagnosis of pharyngitis, a diagnosis of tonsillitis was more likely to result in an antibiotic prescription. This result was consistent with a previous study that found that 71.6% of children diagnosed with acute tonsillitis were prescribed antibiotics, and this far exceeded the standard (0%–20%) set by the European Surveillance of Antimicrobial Consumption Network (ESAC-Net) (23). We postulated that our result was due to the prescribers’ inability to differentiate between bacterial or viral tonsillitis because both cause enlarged and inflamed tonsils. No specific ancillary screening test was done in our ED, such as a rapid streptococcal test to differentiate the causative agents. This may lead prescribers to initiate antibiotic treatment to prevent complications of streptococcal infections.

Ideally, throat swabs should be taken prior to empirical antibiotics; however, due to practical constraints and lack of follow-up in the emergency setting, antibiotics may have been started once a clinical suspicion of streptococcal infection was made. The most common choice of antibiotic is the penicillin group in view of its narrow spectrum against *Streptococcus* species and oral anaerobes, proven efficacy and low cost (24, 25). In our study, both positive and negative McIsaac groups were given broad-spectrum antibiotics (such as amoxicillin and amoxicillin/clavulanic acid) rather than penicillin to treat URTI. This finding was higher compared to a study by Lim et al. (3), which showed that only 2.1% of patients were prescribed broad-spectrum antibiotics in primary healthcare. Although penicillin is effective, compliance to its four-times-daily dosage may have dissuaded prescribers and led them to choose other alternatives that show similar effectiveness (26). Prescribers’ familiarity with the drug may also contribute to this. Another factor may be that the drug is easily accessible to the prescribing doctors, since this study was done in a tertiary hospital. However, this is against our National Antimicrobial Guidelines, which outline the usage of penicillin V or amoxicillin as the first line treatment of URTI for a McIsaac score of more than 3 (27). A recommendation from the Korea Centres for Disease Control and Prevention also states that amoxicillin/clavulanic acid should only be used as second-line therapy in treating URTI if a patient presents with recurrent symptoms (28).

## Limitations

Our study was not without limitations; we may have missed important patient factors that could be associated with inappropriate antibiotic prescribing. This study was a single centre study done in an ED in a tertiary hospital. Therefore, the sample size was small and may not reflect the whole population in the country. Larger studies involving multiple centres can be done in the future to evaluate patient factors. The knowledge and attitude of patients towards antibiotics usage in URTI were also not explored in this study. Therefore, a study to explore these factors could be beneficial. Another potential course of future investigation would be to look at the prescriber factors and laboratory testing that may contribute to inappropriate antibiotic prescribing.

## Conclusion

This study revealed that there is room for improvement with regard to the rate of inappropriate antibiotic prescribing and the choice of antibiotics for URTI in our department. The patient’s symptoms and clinical assessment upon presentation contributed largely to this situation. The factors associated with inappropriate antibiotic prescribing identified in our study include duration of symptoms of one day or less, presence of chills and diagnosis of tonsillitis. Intervention to re-educate, retrain, and provide academic details for the prescribers regarding the importance of a good clinical assessment supported by diagnostic tools such as a scoring system like the McIsaac score in managing URTI may reduce the number of inappropriate antibiotic prescriptions.

## Figures and Tables

**Figure 1 f1-07mjms2802_oa4:**
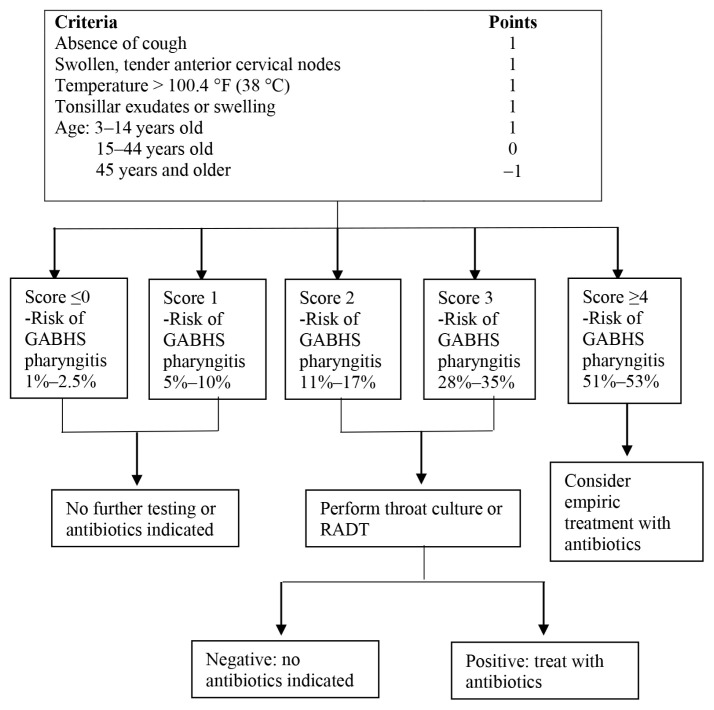
McIsaac scoring management Note: RADT = rapid antigen detection test

**Figure 2 f2-07mjms2802_oa4:**
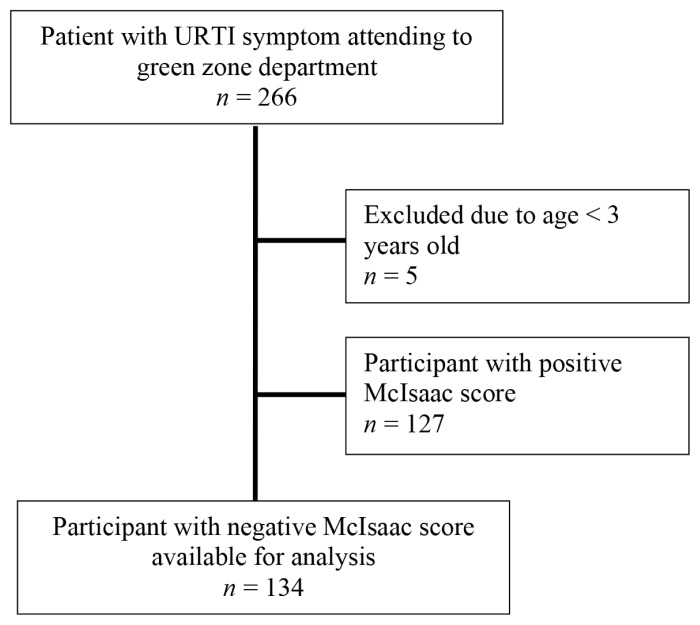
Patient flow diagram

**Table 1:** Demographic characteristics

Variables		Positive McIsaac *n* (%) (*N* = 127)	Negative McIsaac *n* (%) (*N* = 134)
Age, mean ± SD		11.93 ± 11.68	32.54 ± 19.4
Gender	Male	66 (52.0)	80 (59.7)
	Female	61 (48.0)	54 (40.3)
Race	Malay	122 (96.1)	130 (97.0)
	Others	5 (3.9)	4 (3.0)
Working shift	AM	28 (22.0)	36 (26.9)
	PM	57 (44.9)	62 (46.3)
	ON	42 (33.1)	36 (26.9)
Duration of illness	≤ 1 day	17 (13.4)	11 (8.2)
	> 1 day	110 (86.6)	123 (91.8)
Frequency of visits	1st visit	98 (77.2)	106 (79.1)
	2nd visit	28 (22.0)	22 (16.4)
	≥ 3rd visits	1 (0.8)	6 (4.5)
Cough		79 (62.2)	120 (89.6)
Type of cough	Productive	31 (39.2)	59 (49.2)
(if present)	Dry	48 (60.8)	61 (50.8)
Fever		114 (89.8)	110 (82.1)
Sore throat		61 (48.0)	76 (56.7)
Chills		17 (13.4)	16 (11.9)
Muscle pain		14 (11.0)	38 (28.4)
Dyspnoea		3 (2.4)	2 (1.5)
Tachypnoea		2 (1.6)	4 (3.0)
Cervical lymph node	Not palpable	106 (83.4)	118 (88.1)
	Tender	11 (8.7)	3 (2.2)
	Non tender	10 (7.9)	13 (9.7)
Tonsillar enlargement	Absent	28 (22.0)	93 (69.4)
	Exudative	26 (20.5)	8 (6.0)
	Non-Exudative	76 (57.5)	33 (24.6)
Abnormal breath sound*		2 (1.6)	0 (0.00)

Notes: AM = morning shift; PM = evening shift; ON = night shift;

* Abnormal breath sound: including wheezing, stridor and crackles

**Table 2:** Choices of antibiotics given among URTI patients in ED

Variables		Positive McIsaac *n* (%) (*N* = 102)	Negative McIsaac *n* (%) (*N* = 76)
Type of antibiotic	Amoxycillin	49 (48.0)	38 (50.0)
	Erythromycin	0 (0.0)	1 (1.3)
	Amoxicillin/clavulanic acid	53 (52.0)	36 (47.4)
	Cefuroxime	0 (0.0)	1 (1.3)

**Table 3:** Associated factors by simple logistic regression

Variables	Given antibiotic (%) (*n* = 76)	Not given antibiotic (%) (*n* = 58)	Regression coefficient (*b*)	Crude odd ratio (95% CI)	*P*-value
Age			0.01	1.01 (0.99, 1.03)	0.273
Gender					
Male	45 (59.2)	35 (60.3)	0	1	0.894
Female	31 (40.8)	23 (39.7)	0.05	1.05 (0.52, 2.10)	
Frequency of visit					
1st visit	58 (76.3)	48 (82.8)	0	1	
2nd visit	14 (18.4)	8 (13.8)	0.37	1.45 (0.5, 3.74)	0.444
3rd visit	4 (5.3)	2 (3.4)	0.5	1.66 (0.29, 9.43)	0.570
Working Shift					
AM	14 (18.4)	22 (37.9)	0	1	
PM	40 (52.6)	22 (37.9)	1.05	2.84 (1.22, 6.67)	0.044
ON	22 (29.0)	14 (24.2)	0.9	2.46 (0.95, 6.37)	0.062
Fever					
Absent	11 (14.5)	13 (22.4)	0	1	0.238
Present	65 (85.5)	45 (77.6)	0.54	1.71 (0.70, 4.15)	
Diagnosis					
Pharyngitis	40 (52.6)	41 (70.7)	0	1	
Tonsillitis	23 (30.3)	5 (8.6)	1.56	4.72 (163, 13.62)	0.004
Rhinitis	1 (1.3)	7 (12.1)	−1.92	0.15 (0.02, 1.25)	0.078
Tonsillar-pharyngitis	12 (15.8)	5 (8.6)	0.9	2.46 (0.79, 7.62)	0.119
Treating doctor					
House officer	26 (34.2)	22 (37.9)	0	1	
Medical officer	32 (42.1)	24 (41.4)	0.12	1.13 (0.52, 2.45)	0.761
Master student	18 (23.7)	12 (20.7)	0.24	1.27 (0.50, 3.20)	0.613
Cough					
Absent	6 (7.9)	8 (13.8)	0	1	0.274
Present	70 (92.1)	50 (86.2)	0.62	1.87 (0.61, 5.71)	
Rhinitis					
Absent	30 (39.8)	27 (46.7)	0	1	0.412
Present	46 (60.2)	31 (53.3)	0.29	1.34 (0.67, 2.66)	
Sore throat					
Absent	27 (35.5)	31 (53.4)	0	1	0.039
Present	49 (64.5)	27 (46.6)	0.73	2.08 (1.04, 4.19)	
Muscle pain					
Absent	51 (67.1)	44 (75.7)	0	1	0.321
Present	25 (32.9)	14 (24.3)	0.39	1.48 (0.68, 3.20)	
Chills					
Absent	63 (82.9)	55 (94.8)	0	1	0.046
Present	13 (17.1)	3 (5.2)	1.33	3.78 (1.02, 13.97)	
Cervical lymph nodes					
Non-tender	10 (13.2)	2 (3.4)	0	1	
Tender	1 (1.3)	3 (5.2)	1.9	6.67 (0.43, 101.73)	0.172
Not palpable	65 (85.5)	53 (91.4)	0.9	2.45 (0.22, 27.80)	0.469
Tonsillar enlargement					
Non-exudative	19 (25.0)	14 (24.1)	0	1	
Absent	50 (65.8)	43 (74.1)	−0.16	0.86 (0.38, 191)	0.706
Exudate	7 (9.2)	1 (1.8)	1.64	5.16 (0.56, 46.83)	0.145
Duration of illness					
> 1 day	66 (86.8)	57 (98.3)	0	1	0.043
≤ 1 day	10 (13.2)	1 (1.7)	2.16	8.63 (1.07, 69.54)	

Notes: AM = morning shift; PM = evening shift; ON = night shift

**Table 4:** Factors predictive of inappropriate antibiotic prescribing in negative McIsaac score patients by multiple logistic regression analysis (*n* = 134)

Variables		Regression coefficient( *b*)	Adjusted odd ratio (95% CI)	*P*-value
Duration of illness	> 1 day	0	1	
	≤ 1 day	2.92	18.50 (1.65, 207.10)	0.018
Chills	Absent	0	1	
	Present	1.47	4.36 (1.13, 16.88)	0.033
Diagnosis	Pharyngitis	0	1	
	Tonsillitis	1.66	5.26 (1.76, 15.72)	0.003
	Rhinitis	−2.31	0.10 (0.01, 1.32)	0.080
	Tonsillar-pharyngitis	1.06	2.89 (0.91, 9.27)	0.073
